# Animal models for emerging coronavirus: progress and new insights

**DOI:** 10.1080/22221751.2020.1764871

**Published:** 2020-05-13

**Authors:** Lunzhi Yuan, Qiyi Tang, Tong Cheng, Ningshao Xia

**Affiliations:** a State Key Laboratory of Molecular Vaccinology and Molecular Diagnostics, National Institute of Diagnostics and Vaccine Development in Infectious Diseases, School of Life Sciences, School of Public Health, Xiamen University, Xiamen, PR People’s Republic of China; b Department of Microbiology, Howard University College of Medicine, Washington, DC, USA

**Keywords:** Coronavirus, infectious disease, respiratory syndrome, animal model, vaccine and drug discovery

## Abstract

The emergences of coronaviruses have caused a serious global public health problem because their infection in humans caused the severe acute respiratory disease and deaths. The outbreaks of lethal coronaviruses have taken place for three times within recent two decades (SARS-CoV in 2002, MERS-CoV in 2012 and SARS-CoV-2 in 2019). Much more serious than SARS-CoV in 2002, the current SARS-CoV-2 infection has been spreading to more than 213 countries, areas or territories and causing more than two million cases up to date (17 April 2020). Unfortunately, no vaccine and specific anti-coronavirus drugs are available at present time. Current clinical treatment at hand is inadequate to suppress viral replication and inflammation, and reverse organ failure. Intensive research efforts have focused on increasing our understanding of viral biology of SARS-CoV-2, improving antiviral therapy and vaccination strategies. The animal models are important for both the fundamental research and drug discovery of coronavirus. This review aims to summarize the animal models currently available for SARS-CoV and MERS-CoV, and their potential use for the study of SARS-CoV-2. We will discuss the benefits and caveats of these animal models and present critical findings that might guide the fundamental studies and urgent treatment of SARS-CoV-2-caused diseases.

## Introduction

The pleomorphic and enveloped coronaviruses are a genus in the *Coronaviridae* family and contain a ∼30 Kb positive-sense RNA genome [1]. The viral envelop contains envelope (E) protein, transmembrane (M) and spike (S) glycoprotein, and surrounds a disordered or flexible nucleocapsid (N) [2]. Most coronaviruses infect wild animals with narrow host range and cause self-limiting diseases [1]. Human coronaviruses such as OC43, 229E, NL63 and HKU1 are associated with self-limiting respiratory tract infections [3]. However, two zoonotic coronaviruses, the severe acute respiratory syndrome coronavirus (SARS-CoV) and middle east respiratory syndrome coronavirus (MERS-CoV), cross the species barrier to infect humans and have caused severe acute respiratory disease (SARD) and thousands of deaths [4,5]. Since December of 2019, a new “SARS-like” coronavirus, named SARS-CoV-2, raised intense concerns not only within China but also internationally [6]. Importantly, SARS-CoV-2 showed approximately 80∼90% of genome sequence homology with previously identified SARS-CoV strains, suggesting an evolving similarity in virological properties [7].

Receptor-mediated entry is the first step of a viral infection in the host cell. Receptor binding domain (RBD) of the viral S protein of SARS-CoV or MERS-CoV attaches to human angiotensin converting enzyme 2 (hACE2) or dipeptidyl peptidase 4 (hDPP4) proteins, respectively. The hACE2 has been also believed to be the receptor of SARS-CoV-2 [7]. After entry into cells, these three coronaviruses (SARS-CoV, MERS-CoV and SARS-CoV-2) replicate efficiently to produce progeny viruses, give rise to cytopathogenesis and establish productive infection. SARS-CoV of 2002 caused over 8098 cases and 774 deaths in over 30 countries. MERS-CoV of 2012 resulted in more than 2182 cases and 779 deaths in 27 countries. SARS-CoV-2 has caused over two million cases in more than 213 countries, areas or territories with over 150,000 deaths up to date (17 April 2020). Therefore, it is highly emergent to obtain the effective clinical medication and vaccines to prevent and treat coronavirus infection.

**Figure 1.**
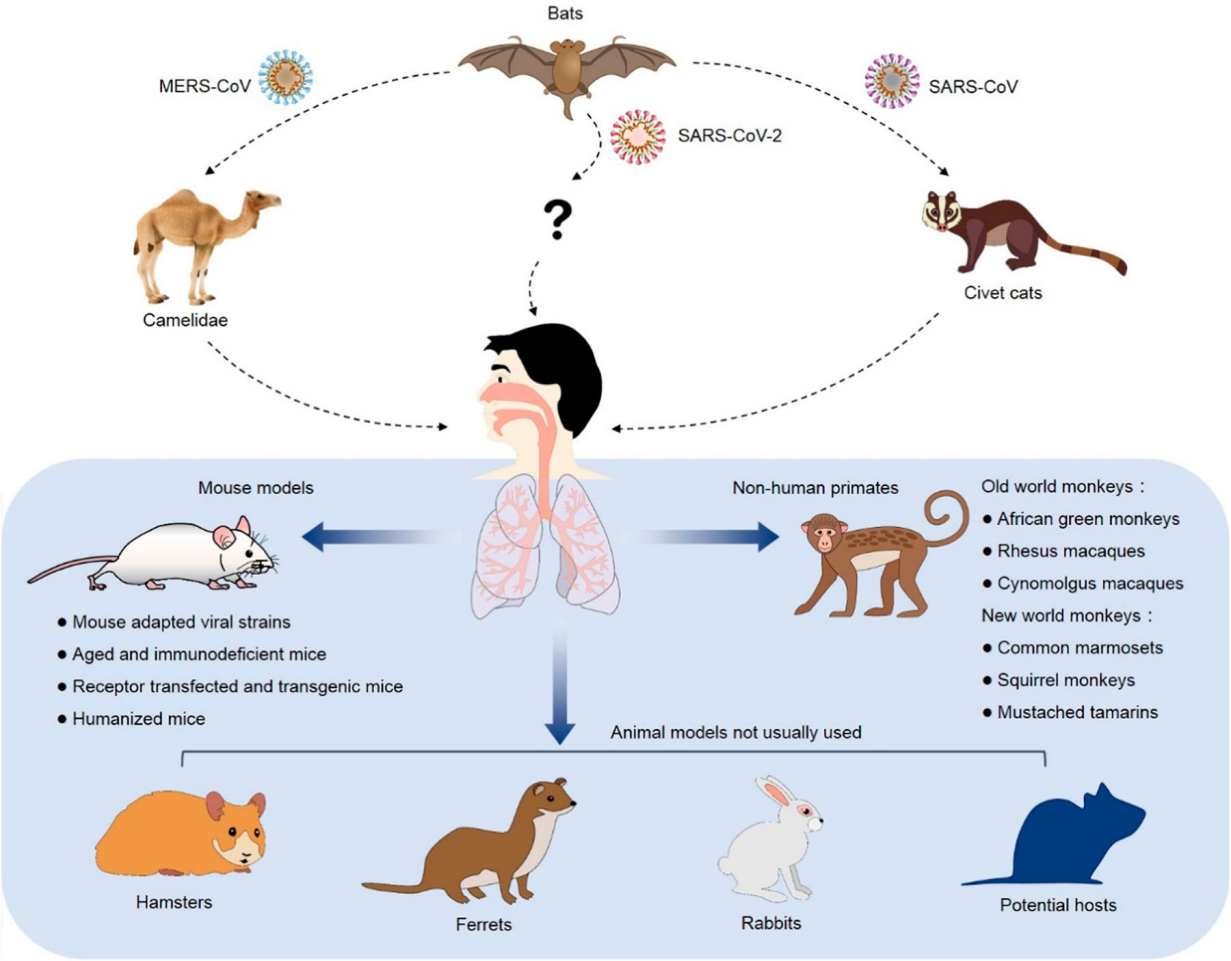
Experimental animals of SARS-CoV, MERS-CoV and SARS-CoV-2. The coronaviruses with high infectivity and pathogenicity break the species barrier and infect human in the past two decades. Besides NHP, mice, hamsters, ferrets and rabbits, the other possible natural hosts might be able to support the studies of coronavirus infection, pathogenesis and drug discovery.

Animal models are critical for us to understand the viral infection and pathogenesis. Moreover, animal models are essential for development and preclinical evaluation of a vaccine or an antiviral agent. An ideal animal model is the one that mimics viral infection and diseases in humans in multiple aspects including morbidity, viral load, typical clinical symptoms, host immune responses and mortality. Therefore, the urgent need of preventing and controlling coronavirus infection necessitates the search for an optimal SARS-CoV-2 animal model. Based on the published studies, animal models of SARS-CoV and MERS-CoV include civet cats, camelidaes, monkeys, mice, hamsters, ferrets, rabbits and other potential hosts (Figure 1). We aim to summarize and discuss their ability to mimic the disease symptoms and natural history of coronavirus disease 19 (COVID-19) in humans, as well as their usage in development of vaccine and antiviral drugs. Furthermore, humanized animal models available to support coronavirus infection and pathogenesis might provide new options to overcome the limitations of the traditional coronavirus animal models. Additionally, animal models for pseudovirus are also prospected to avoid the concern of biosafety.

## Emerging coronaviruses infection in humans

Clinical symptoms of SARS-CoV- and MERS-CoV-infected patients at early time include fever, chills, coughing, malaise, myalgia, headache, diarrhoea, vomiting and nausea [8]. Furthermore, immunohistochemistry (IHC) detection demonstrated the presence of viral antigens in lung tissues. The COVID-19 patients present similar symptoms to those of SARS-CoV- or MERS-CoV-infected patients, while some patients may show no typical clinical symptom in the early stage of infection [9]. The typical pathological features of severe cases include prolonged inflammation with destruction and desquamation of alveolar pneumocytes, hyaline-membrane formation, interstitial inflammatory infiltration and interalveolar hemorrhage. Multinucleated giant cells were also observed in the tissues of COVID-19 patients [10]. Over 70% of COVID-19 patients were diagnosed as pneumonia by chest computed tomography (CT) to be admitted to hospital. CT images showed the typical features of ground-glass opacity and bilateral patchy shadowing in lungs [9]. Recent clinical and experimental studies have demonstrated SARS-COV-2 caused the nosocomial infection and fecal-oral transmission, its infection results in immune abnormality and multiple organ failures of the COVID-19 patients. Moreover, a long incubation period (>29 days) was observed in some COVID-19 cases [11], suggesting a risk of occult and chronic infection. Although SARS-COV-2 displays a lower rate of severe cases and case fatality than SARS-CoV and MERS-CoV, its high infectivity, widely spreading and huge infection population have become a catastrophic medical burden and critical social problem.

## Natural infectious animal models

Viral entry and intracellular replication are two essential steps for a virus to establish a successful infection. The specific receptor proteins anchored on cell membrane mediate viral entry and intranuclear transcriptional factors regulate viral replication. Therefore, host-range of virus depends on these two steps. As summarized in Table 1, non-human primates (NHPs), ferrets and hamsters have been demonstrated to support SARS-CoV infection and pathogenesis, but only the NHPs are fully permissive for MERS-CoV infection. The civet cats were demonstrated permissive for SARS-CoV infection by serological antibody detection. The camelidaes have been demonstrated permissive for MERS-CoV infection because the specific antibodies against MERS-CoV S protein were detected at a high rate, and respiratory infections after the administration of MERS-CoV. Immunocompetent young inbred mice support transient SARS-CoV infection without clinical signs of disease [12], but were not permissive to MERS-CoV due to the lack of functional receptor [13]. After a careful review of their illness, virological, histological and immunological characteristics post infection, NHPs, ferrets and hamsters have been presumed to be potential natural hosts of SARS-CoV-2. Meanwhile, other experimental animal models such as tree shrew, woodchuck, pangolin, rat, guinea pig and cotton mouse, as well as domesticated animals such as cats [14] and dogs might be potential hosts to support SARS-CoV-2 infection.

**Table 1.** Natural infectious animal model for SARS-CoV and MERS-CoV.

Animals	SARS-CoV	MERS-CoV
NHPs	Rhesus and cynomolgus macaques, African green monkeys, common marmosets, squirrel monkeys and mustached tamarins; clinical signs, viral replication and pathology degree varied on the species	Common marmosets have a more severe response to the virus with higher viral titres and severe pathology than rhesus macaques; lethality is only observed in common marmosets
Mice	Immunocompetent young inbred mice support transient infection without clinical illness	Not permissive for entry
Hamsters	Self-limiting infection; mild clinical illness	Not permissive for viral replication
Ferrets		
Civet cats	Permissive to infection and replication; mild clinical illness	No report
Rabbits	No report	Infection with mild clinical illness
Camelidae	No report	Infection without clinical illness

### The NHPs

The studies of coronavirus have been greatly benefited from the using NHPs as the infection and disease models. The old- and new-world monkeys include rhesus macaques *(Macaca mulatta)*, cynomolgus macaques *(Macaca fascicularis)*, African green monkeys *(Chlorocebus sabaeus)*, common marmosets *(Callithrix jacchus)*, squirrel monkeys *(Saimiri)*, and mustached tamarins *(Saguinus mystax)*, all of them have been demonstrated to be permissive for SARS-CoV infection to cause symptoms such as fever, diarrhea and clinical manifestations of pneumonitis [15,16]. The African green monkeys, cynomolgus and rhesus macaques showed mild symptoms after infection of SARS-CoV Urbani strain and they produced neutralizing antibodies (NAbs) that might help to clear the virus [17]. In another study on rhesus macaques challenged with the SARS-CoV PUMC01 strain, transient severe pneumonia was observed [18]. Meanwhile, Greenough et al. reported SARD with fever, diarrhea, hepatitis and multi-organ failure in common marmosets around four to seven days post SARS-CoV infection [19].

In some studies, the rhesus macaques and common marmosets were used as a model for MERS-CoV infection. However, MERS-CoV infections in the two NHPs caused different levels of consequences. First, the symptoms in the common marmoset model was much more severe and viral yield was approximately thousand-fold higher than those in the rhesus macaque model. Secondly, viral replication in the common marmosets reached peak level and the viral infection caused severe pneumonia within four to six days post MERS-CoV infection [20]. However, MERS-CoV infection in the rhesus macaques did not produce a high viral load and didn't cause severe symptoms [21]. The rhesus macaque has been demonstrated permissive for SARS-CoV-2 infection via ocular conjunctival route [22]. SARS-CoV-2 induced a transient infection and mild pneumonia in the rhesus macaques. However, severe pneumonia and death were not observed. Meanwhile, the infected rhesus macaques pounced NAbs that effectively prevent re-challenge of SARS-CoV-2 [23]. More recently, the cynomolgus macaques and common marmosets were also demonstrated available to support SARS-CoV-2 infection [24]. In this study, rhesus macaques were demonstrated more susceptible to SARS-CoV2 infection as compared to cynomolgus macaques and common marmosets.

### Ferrets, hamsters and rabbits

Ferrets has been used as a model for the studies of respiratory pathogens including influenza virus (IFV), respiratory syncytial virus (RSV), adenovirus and coronavirus. Up on infection of SARS-CoV, the ferret presented fever and sneezing associated with detectable viral titres in the upper respiratory tract [25]. However, severe symptoms and deaths caused by viral infection were not observed, and ferrets do not support replication of MERS-CoV [26]. Recently, ferrets were demonstrated available to support SARS-CoV-2 infection by two different groups [14,27]. It was reported that SARS-CoV infects the golden Syrian hamster [28]. Viruses seeded in the upper and lower respiratory tract and reached a peak level at three days post SARS-CoV infection and declined and cleared within seven to ten days. The infection of SARS-CoV resulted in mild and transient pneumonia. By contrast, similar infection course and symptoms were recently observed in hamsters that infected with SARS-CoV-2 [29]. Hamsters have been demonstrated not susceptible to MERS-CoV infection [28]. Interestingly, primary SARS-CoV and SARS-CoV-2 infection in ferrets and hamsters elicited a strong NAb response that protected the hosts from subsequent infections. The above-mentioned information implies that SARS-CoV-2 infection might induce a mild to severe pulmonary diseases in ferrets and hamsters. The severities of symptom might depend on viral strains. The ability of MERS-CoV to infect small animal species may be defected by lack of viral entry or replication. Surprisingly, rabbits were susceptible to MERS-CoV infection [30]. Although viral nucleic acid and infectious progeny virus were detectable in lung tissues, neither significant histopathological changes nor clinical symptoms were observed. These findings indicate a potential route of MERS-CoV transmission in some common animal species. However, there is no reported investigation of SARS-CoV and SARS-CoV-2 infection in the rabbits. Therefore, it is important to experimentally investigate whether ferret, hamster and rabbit are susceptible to the infection and are able to be used as animal model of SARS-CoV-2.

## Mouse models

Mouse model has been widely used for many different viral investigations. It has been considered as the best small animal model for hepatitis B virus (HBV), hepatitis C virus (HCV), cytomegalovirus (CMV), Zika virus, and among others. Due to its low cost, small size, easy operation and high reproducibility, mouse model is suitable for large scale studies of viruses not only for the pathogenesis but also for antivirals. In contrast of the NHPs, mice are more convenient to be handled in a higher level of biosafety laboratory due to its relatively small size and less operation difficulties. Importantly, mouse can be easily manipulated at the genetic level for precision research. For instance, a lot of genetically mutated mice are available for studies in anti-viral immunity, viral pathogenesis and viral infection and transmission restriction. Furthermore, current murine immunological reagents are available to study viral pathogenesis and host immune responses.

### Mouse expressing human receptor

Several strains of mice have been tried to be intranasally infected with SARS-CoV (Urbani strain), it was found that the virus poorly replicated in several young and adult inbred strains of mice (BALB/c, C57BL6 and 129S) to only a low viral yield. Clinical illness was not observed, and the virus was cleared within nine days. Meanwhile, mice are not naturally susceptible to MERS-CoV infection. It was later revealed that the mouse DPP4 receptor differs from human counterpart in crucial domains that is critical to bind the S protein [13]. To overcome the species-specificity barrier for SARS-CoV or MERS-CoV, human receptor protein (hACE2 or hDPP4) was expressed in mice by knocking in or transfecting a human receptor gene in mouse. The studies of hACE2-transgenic mice were performed at almost the same time in 2007 independently in three groups. They reported that hACE2-transgenic mice supported SARS-CoV infection and pathogenesis. McCray et al. demonstrated that systemic expression of hACE2 under the control of an epithelial cell-specific promoter K18 resulted in lethal SARS-CoV infection within five days [31]. Tseng et al. developed two lineages of transgenic mice expressing hACE2 under the CAG promoter [32]. After SARS-CoV infection, the two transgene-positive mice, AC70 and AC63 showed high viral load, clinical illness and tissue pathology. The lethal lineage of mice (AC70) displayed higher hACE2 expressions in several organs, wider spectrum of clinical illness, including wasting symptom, severe pneumonia and death, rather than the non-lethal lineage mice (AC63) with lower hACE2 expressions. Yang et al. generated another non-lethal lineage mouse that expresses hACE2 under mouse ACE2 promoter [33]. Another study further revealed the differential virological and immunological outcomes among different hACE2 transgenic lineages [34].

To rapidly generate a MERS-CoV infecting mouse model, Zhao et al. transduced common adult BALB/c and C57BL6 mice with an adenoviral vector expressing hDPP4 (Ad5-hDPP4). After MERS-CoV infection, these mice showed interstitial pneumonia, expressed viral antigen in the lungs and lost weight, but deaths were not observed. Later, hDPP4-transgenic mice were generated to mimic severe MERS-CoV infection and lethal pathogenesis. Agrawal et al. developed a transgenic mouse that expresses hDPP4 under the control of the CAG promoter [35]. This hDPP4-transgenic mouse was fully permissive for MERS-CoV infection to cause severe respiratory illness that led to death within six days after infection. Remarkably, high viral load was detected in multiple organs and pathological changes were consistent with extensive inflammation throughout the infection course. In addition, MERS-CoV infection induced profound acute innate inflammatory responses within the lungs of this model. In 2017, Li et al. developed another hDPP4-transgenic mouse and demonstrated that MERS-CoV infection induced lethal lung disease is viral load dependent [36]. Similar to the model by Agrawal et al., activated innate immune cells accumulated in the lungs after MERS-CoV infection. Recently, Yoshikawa et al. observed the cytokine storm in a newly generated hDPP4-transgenic mouse [37].

These receptor-transgenic or -transduced mice have been widely used to study the pathogenesis mechanism and evaluate vaccines and other therapeutics (Table 2). Because hACE2 has been recently demonstrated as the receptor of SARS-CoV-2 in a cell infectious model [7], the hACE2 transgenic mice are supposed as an option to study SARS-CoV-2 infection and pathogenesis. Recently, Bao et al demonstrated that hACE2-transgenic mice are able to support SARS-CoV-2 infection [38]. It is observed that mice presented weight loss, virus replication, infiltration of lymphocytes and monocytes in alveolar interstitium and accumulation of macrophages in alveolar cavities from three to five days post SARS-CoV-2 infection. However, no significant histopathological lesions or viral antigens were observed in the other organs, including myocardium, liver, spleen, kidney, cerebrum, intestine, and testis. Furthermore, pneumonia became mild with focal lesion areas at seven days post infection, suggesting a non-lethal and self-limiting infection course. This lineage of hACE2-transgenic mouse cannot mimic the severe and lethal cases of COVID-19, which necessity attempts in other lineages with different hACE2 promotors. Alternatively, transduction of Ad-hACE2 in young adult BALB/c and C57BL6 mice might provide an avenue for rapid and robust generation of SARS-CoV-2 infectious mouse model.

**Table 2.** Representative receptor transgenic and transfected mice support SARS-CoV and MERS-CoV infection.

	SARS-CoV		MERS-CoV			
	McCray et al. [31]	Tseng et al. [32]	Zhao et al. 2014	Agrawal et al. [35]	Li et al. [36]	Iwata-Yoshikawa et al. [37]
Background	C57BL6		C57BL6; BALB/c	C57BL6		BDF1 × C57BL6
Age (weeks)	6∼8	8∼20	6∼22	5∼7	6∼13	9∼25
Viral strains	Urbani		EMC/2012; EMC/Vero; MERS_MA_	EMC/2012; rMERS-CoV/RFP	EMC/2012; EMC/Vero; MERS_MA_	EMC/2012;
Inoculation dose	2.3 × 10^5^ PFU	2 × 10^5^ TCID_50_	1 × 10^5^ PFU	1 × 10^6^ TCID_50_	50∼5000 PFU	1 × 10^5^ TCID_50_
Clinical illness	Severe pneumonia; weight loss; robust cytokine storm in lung and brain		Mild inflammatory cell infiltrates; weight loss	Severe pneumonia; weight loss; robust cytokine storm in lung and brain	Viral strain dependent; mild to severe pneumonia; weight loss; activation of innate immune cells	Mild pneumonia; weight loss; slight cytokine storm in lung and brain
Peak viral load	∼10^8^ TCID_50_/g in lung at 4dpi	∼10^9^ TCID_50_/g in lung and brain at 4dpi	∼10^7^ PFU/g in lung at 2∼3dpi	∼10^8^ TCID_50_/g in lung at 2dpi; ∼10^5^ TCID_50_/g in brain at 4dpi;	Viral strain dependent; ∼10^8^ PFU/g in lung at 1∼3dpi (5000 PFU of MERS_MA_)	∼10^5^ TCID_50_/g in lung at 3dpi
Mortality rate	∼90% at 4 dpi	AC63 (None); AC70 (100% at 8 dpi)	None	100% at 6 dpi	Viral strain and inoculated dose dependent; 100% at 6 dpi (5000 PFU of MERS_MA_)	None

### Adapting human coronavirus to mouse

**Figure 2.**
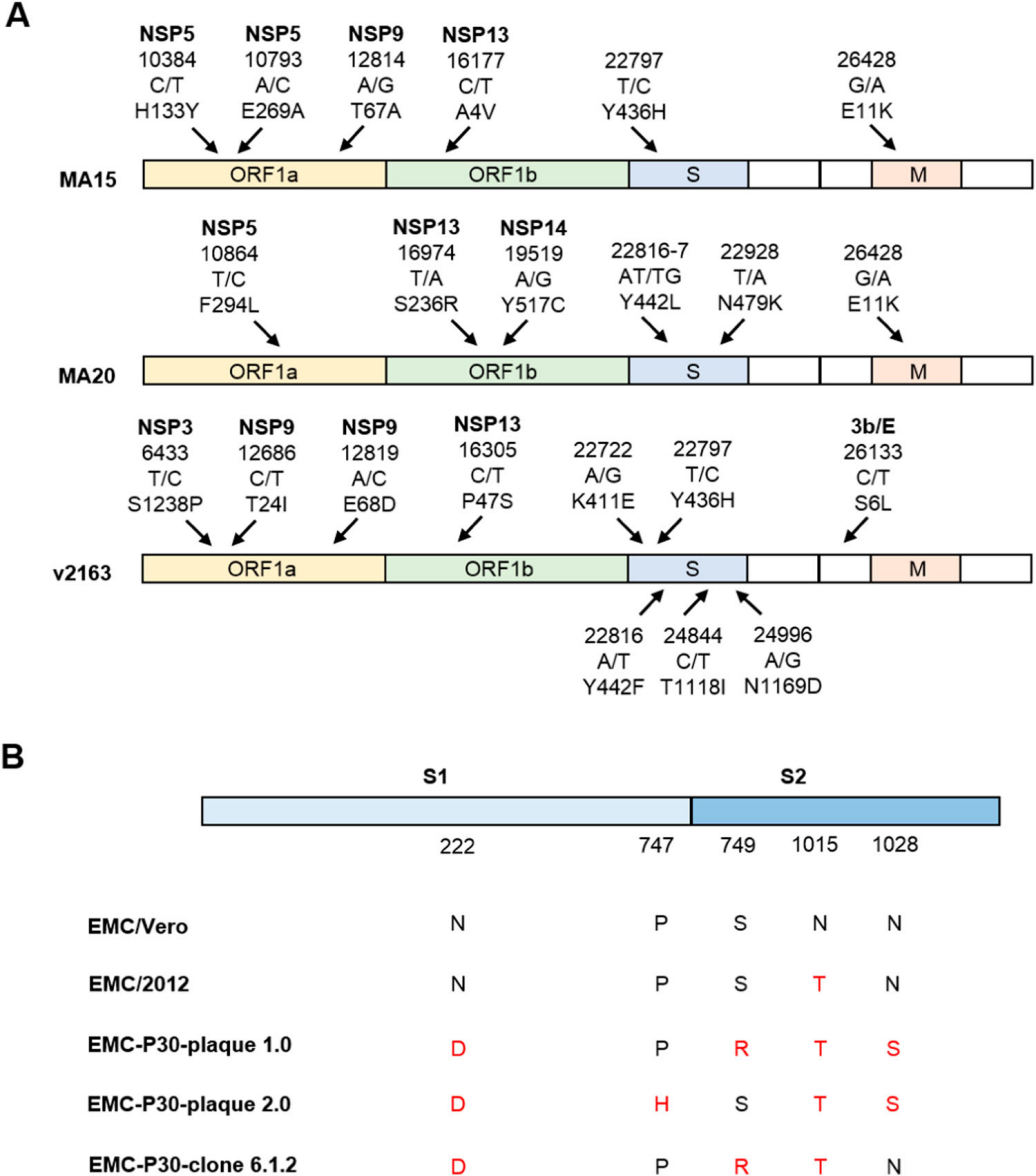
Mutations in MA stains of SARS-CoV and MERS-CoV. (A) Nucleic acid and amino acid mutations of SARS_MA_ strain MA15, MA20, and v2163 [39]. (B) Amino acid changes of MERS-CoV S1 (receptor binding) and S2 (fusion) domains of different plaques and clones of the EMC-P30 strain [36].

In order to promote the replication of SARS-CoV in young adult immunocompetent mouse, three mouse-adapted (MA) viral strains were developed by serial passage of SARS-CoV (Urbani strain) in mouse respiratory tract. In contrast to the original Urbani strain, the SARS-CoV MA (SARS_MA_) strains such as MA15, MA20 and v2163 replicated in the lungs of mice to a higher titre that is proportionally associated with pathological changes, dissemination of the virus to extrapulmonary sites and mortality (Table 3). As shown in Figure 2A, several mutations occurred in ORF1a (nsp5 and nsp9), ORF1b (nsp13), and S protein. It is believed that the combination of these genetic changes enhanced the viral virulence [39], implying the importance of these proteins in viral pathogenesis. In brief, the three SARS_MA_ strains induced SARD in mice that is similar to the clinical symptoms observed in human cases of SARS-CoV infection.

**Table 3.** Infection outcomes of mouse-adapted SARS-CoV strains.

Strains	Urbani (wt)	Urbani_+S_	Urbani_+N9_	Urbani_+N9/S_	MA15	MA_+N9/S_	MA20	v2163	Urbani	MA15
Mouse	One-year-old BALB/c						5∼10 week BALB/c			
Inoculation dose	1 × 10^5^ PFU								1 × 10^5.5^ CCID_50_	
Peak viral load in lung at 2∼4 dpi	∼10^5^ PFU/g	∼10^7.2^ PFU/g	∼10^8^ PFU/g	∼10^7.5^ PFU/g	∼10^6^ PFU/g	∼10^6.5^ PFU/g	∼10^6.5^ PFU/g	∼10^7.5^ PFU/g	∼10^5.8^ CCID_50_/g	∼10^6.6^ CCID_50_/g
Mortality rate	None	100% at 8dpi	None	100% at 4dpi	100% at 5dpi	100% at 4dpi	ND	100% at 6dpi	None	90% at10 dpi
Mean day of death (days)	–	6.0 ± 1.26	–	3.2 ± 0.44	3.8 ± 1.09	3.2 ± 0.40	ND	4.8 ± 1.8	–	6.8 ± 1.4
LD_50_	–	10E4.4	–	10E2.5	<10E2	10E2.3	ND	10E3.3	–	–

Ferrets, hamsters and mice are not naturally susceptible to MERS-CoV infection, because MERS-CoV S protein cannot bind DPP4 of these animals. Thus, progeny infectious virus could not be isolated from these animals after initial infection, and the MERS-CoV adaptation is unlikely to be carried out. As mentioned above, MERS-CoV can successfully infect a hDPP4-transgenic mouse. Interestingly, *in vivo* serial passage of the EMC/2012 strain of MERS-CoV in the hDPP4 knock-in mice resulted in a MA virus strain (MERS_MA_) that causes a fatal pulmonary disease phenotype associated with severe inflammatory responses [36]. Furthermore, it was reported that more passages in the mouse caused more severe diseases. For example, the MERS_MA_ strain with 21 adaptive passages (EMC-P21) cause 50% lethality of hDPP4 transgenic mice, while the strain with 30 adaptive passages (EMC-P30) killed all the mice within eight days [36]. Moreover, different mutations in MERS-CoV S1 (receptor binding domain) and S2 (fusion domain) occur in different plaques or clones of the passaged EMC-P30 strain, implying that the gene diversity has impacts on the viral entry (Figure 2B).

The S protein of coronaviruses needs to be cleaved by host proteases to be functional. The proteases include endosomal cathepsins, cell surface transmembrane protease/serine (TMPRSS) proteases, furin, and trypsin [40]. For example, SARS-CoV-2 uses serine protease, TMPRSS2 in host cell to cleave S protein to activate its entry process [41]. The S protein mutations enhanced infectivity of MERS_MA_ strains by promoting early entry and broadening tissue tropism [42]. Comparing to MERS strain with an uncleaved S protein, MERS_MA_ strain has an S protein that is pre-cleaved by furin of the infected cells and hence achieves a non-endosome cellular entry. Thus, mutations in the protease cleaving site of S protein might alter infectivity of SARS-CoV-2. Considering the similarity of receptor and highly gene sequence homology between SARS-CoV and SARS-CoV-2, adaptive viral strain of the novel coronavirus might be obtained after 10∼20 serial passage in hACE2 transfected cells or animal models. Overall, generating adaptive viral strains might become another approach to enhance the infectivity and establish an effective and lethal SARS-CoV-2 infection in experimental animals.

### Immunodeficient mouse

Several studies have attempted to use the immune-compromised mice to determine the role of immune effectors in the coronavirus infection. First, the following knock-out mice have been applied for the infection of SARS-CoV: beige lacking functional NK cells, CD1^−/−^ lacking NK-T cells, Rag^−/−^ lacking T and B cells [43]. However, all the immune-compromised mice failed to allow the infection of SARS-CoV. Viral kinetics were not significantly different among the 57BL6 (wild type), beige, CD1^−/−^ and Rag1^−/−^ mice. Histopathological detection showed similar self-limiting bronchiolitis and mild pneumonia among these mice. Interestingly, a prolonged viral replication and illness were observed in STAT1^−/−^ mice with 129S background [44]. In this model, viral replication is detectable until day 22 post infection indicating that a STAT1 mediated type I interferon response is required to control SARS-CoV infection [45]. The STAT1^−/−^ mice were also challenged with MERS-CoV (EMC-2012 strain). Because the lack of receptor hDPP4, clinical illness were not observed and viral replication was undetectable [46]. However, the hDPP4-transfected mice additional deficiency of interferon-α receptor, MyD88 and MVAS still cannot prolong the viral replication or cause severe cause throughout a MERS-CoV infection course [36]. Generally, mice with targeted immune deficiency are useful tools to investigate interaction between host immunity and coronavirus. Besides the relatively low viral load and mild illness, the immunodeficient mice are of limited value in the studies of vaccine and immunotherapy.

### Host factors impact on coronavirus infection

It is important to know the risk factors that affect the clinical outcomes of coronavirus infection [47]. First, does age play an important role in disease severity? To know that, an aged mouse model was used for SARS-CoV infection and it was found that SARS-CoV infection in aged mice resulted in increased mortality than that in younger mice. In the studies, 12–14 month old BALB/c or C57BL6 mice developed more severe disease include viral replication, weight loss, ruffled fur and dehydration than that in the 6∼12 week-old ones [48]. However, aged 129SvEv mice did not support prolonged pulmonary viral replication and cleared the virus within five days [49]. Interestingly, the SARS_MA_ strain MA15 showed higher mortality in the 9∼20 month aged BALB/c and C57BL6 mice than that in the 2∼5 month ones [50]. Recently, Yoshikawa et al. demonstrated that the immunopathology degree of MERS-CoV infection in the hDPP4-transgenic mouse is age dependent [37]. Mechanistic studies demonstrated that age-dependent increases of phospholipase PLA_2_G2D and dysfunction of CD4 positive T cell could be the reasons for the enhanced SARS-CoV infection [51,52]. According to epidemiology data, SARS-CoV-2 is more likely to cause severe causes and high mortality in elder people [53]. Overall, the aged mouse model provided an opportunity to study the age-dependent susceptibility of humans to coronavirus.

Second, does the gender has impact on infection outcomes? Generally, the males generate mild immune responses and females mount stronger innate and adaptive immune responses and are relatively resistant to virus infections, which explains why males and females showed different response patterns after infection of viruses. Recent studies showed the higher expression of hACE2 in Asian males than the females [54]. Epidemiological data showed gender-specific differences in SARS-CoV infection with males experiencing higher mortality compared to females [55]. This gender-dependent increase in disease severity after pathogenic SARS-CoV infection was more pronounced with advancing age [55]. Male C57BL6 and BALB/c mice showed more susceptible to and higher mortality after SARS-CoV MA15 infection than female mice [50]. In addition, the male mice displayed a higher proinflammatory cytokine levels than female ones, which was is independent of T and B cell responses throughout the SARS-CoV infection course. These clinical and experimental findings imply a higher risk of SARS-CoV and SARS-CoV-2 infection in the elder Asian males. However, MERS-CoV infection showed no difference between male and female hDPP4-transgenic mice in mortality and weight loss. The epidemiological data of MERS-CoV infection in human with different gender is conflict. Some study showed high incidence and mortality among the males [56], and others showed that gender may not have impact on the infection outcome of MERS-CoV [5]. Thus, it is necessary to clarify the conflicting results of gender impacts in clinical and experimental studies of MERS-CoV infection. Overall, gender might be an interesting topic for further investigation for the mechanism of SARS-CoV-2 infection and pathogenesis. In addition, infection routes and inoculation doses might also have impact on the characteristics of virus shedding, pathogenesis and disease outcomes in experimental animals.

Presumptively, animal models of SARS-CoV might fit for the study of SARS-CoV-2 due to the similarities of the two viruses in pathogenesis, replication, viral characteristics and host receptor for viral entry. Therefore, combination of hACE2 transgenic- or transduce-mice and SARS-CoV-2 MA strains might result in an enhanced infection, severe pathogenesis and higher mortality, which resembling the clinical illness. Comparing to SARS-CoV and MERS-CoV, the SARS-CoV-2 might be more complicated in transmission and pathogenesis, which makes the animal studies more difficult. For example, SARS-CoV-2 infection has a long incubation time [11], more rapid transmission among populations [53] and more severe multiple organ failure [47].

## Coronavirus vaccine candidates evaluated in animal models

An appropriate animal model is important to evaluate a vaccine in medical safety, protective efficacy and comprehensive immune activation. Although many attempts of developing vaccines have been tried, there is currently no approved vaccine for SARS-CoV and MERS-CoV. Several approaches to develop SARS-CoV and MERS-CoV vaccines were evaluated in indicated animal models that are summarized in Table 4. The traditional inactivated virus vaccines use chemicals or radiation to render the viral genome non-infectious while maintaining the virion structure, thus preserving antigenicity but eliminating the potential to cause productive infection. Animal studies have assessed the inactivated SARS-CoV vaccines and found that they can elicit the production of NAbs and inhibit viral replication in NHPs, mice, ferrets and hamsters. However, in some cases, the inactivated vaccine may not arouse enough NAbs for the cover or damage of dominant epitopes [57]. Moreover, the inactivated vaccines might lack adequate cross-protection among different viral strains, especially for the unknown and newly identified strains. Live-attenuated vaccines are produced by reducing or eliminating the virulence of a live virus, typically using chemical-driven, site mutation or gene deletion. Although the live-attenuated vaccine can elicit both innate and adaptive immune responses in mice and hamsters, risk of phenotypic or genotypic reversion and disseminated infection in immunocompromised patients limit further translation [1].

**Table 4.** Experimental animals for development of SARS-CoV and MERS-CoV vaccines.

Vaccine type	Design strategy	Animal models and examples		Advantages	Disadvantages
		SARS-CoV	MERS-CoV		
Inactivated particle	Whole virus inactivated by heat, chemicals, or radiation	African green monkeys, rhesus and cynomolgus macaques; BALB/c, C57B6 and 129S6/SvEv mice; ferrets; rabbits	BALB/c mice	Rapid and easy for development; safety; high-titre NAbs; protective when used with adjuvant	Induction of inflammatory immune pathology and ADE; possibly incomplete protection
					
					
					
Live-attenuated particle	Attenuated the virulence by viral genome mutagenesis or targeted deletions	BALB/c mice; hACE2-Tg mice; hamsters	BALB/c mice	Inexpensive; quick immunity; less adverse effect; comprehensive activation of host immunity; multiple targets	Risk of phenotypic or genotypic reversion and disseminated infection in immunocompromised patients
					
					
					
Vector	Viral vectors that express S or the S1 subunit	BALB/c and 129S6/SvEv mice; hamsters; ferrets	BALB/c mice; hDPP4-Tg or transfected mice; dromedary camels	Comprehensive, Stronger and specific activation of host immunity; high-titre NAbs; safety	Varied and skewed immune responses; possibly incomplete protection
					
					
Virus-like particle	Virus-like replicon particles containing S protein	BALB/c mice	Ad-hDPP4 mice; BALB/c mice	Induction of antiviral T cell responses and S protein specific NAbs; reduce viral titres in lungs to nearly undetectable levels by one after inoculation with MERS-CoV	Varied and skewed immune responses; risk of ADE; possibly incomplete protection
Nanoparticle	Purified S protein-containing nanoparticles; Gold nanoparticle adjuvanted S protein	BALB/c mice	BALB/c mice	Induction of S protein specific NAbs	Need appropriate adjuvants; Varied and skewed immune responses; risk of ADE
Viral subunit	Antigenic components, whole or separate (S and N protein)	Rhesus macaques, BALB/c mice and rabbits	Rhesus macaques; hDPP4 transgenic mice; BALB/c mice	High safety; consistent production; Comprehensive, Stronger and specific activation of host immunity; high-titre NAbs	Uncertain cost-effectiveness; mild immunogenicity; need appropriate adjuvants; risk of ADE
					
					
DNA plasmid	DNA plasmids that encode S and N protein	BALB/c mice	Rhesus macaques and BALB/c mice; Ad-hDPP4 mice	Easier to design; high safety; high-titre NAbs	Lower and skewed immune responses; possibly delayed-type hypersensitivity
					
					

Among all the functional structural proteins of coronavirus, the S protein is an important antigen that might effectively inducing NAbs to block viral binding and entry, as well as stimulating robust host immune responses [58]. Therefore, the S protein has been designed as the main target for the development of SARS vaccines by different strategies including developing a S subunit, using an expression vector, packaging a virus-like particle, delivering a DNA plasmid and nanoparticle [3]. These vaccine strategies showed protection effects in different animals (Table 4). However, they are limited by the possibilities of incomplete protection, the risk of antibody dependent enhancement (ADE) include expanded infection, tissue destruction, cytokine storm and multiple organ failure [3]. The mechanism of ADE effect is complicated and not yet clearly clarified. Serum of inactivated SARS-CoV and S protein immunized mice, as well as the S protein specific antibodies were demonstrated to promote viral infection and cytopathogenesis in human immune cells such as macrophage and B lymphocytes, via a pH- and cysteine protease-independent Fcγ receptor pathway [59]. Moreover, S protein specific antibodies treatment caused fatal acute lung injury in SARS-CoV infected NHPs by skewing macrophage mediated inflammation-resolving responses [60]. In addition, prior immunization with N protein might also cause severe pneumonia in mice infected with live SARS-CoV [61]. Indeed, many studies showed that virus-like particle, inactivated virus vaccine, rDNA-produced S protein vaccine led to occurrence of pulmonary and Th2-type immunopathology on challenge with live SARS-CoV in mice, suggesting hypersensitivity to SARS-CoV components was induced [57,62]. Similar lung immunopathology was also observed in mice immunized with inactivated MERS-CoV vaccine [63]. These findings suggested that a potential pathogenic effect of antibodies targeted at SARS-CoV-2 would be of major concern for vaccine development and antibody-based therapies.

Although the NHPs were supposed to be closer to human, different mouse models and MA viral strains have occupied a large proportion of these coronavirus vaccine studies for their advantages of convent and large-scale to draw a more robust conclusion. Due to the genetic differences, the results from animals are not necessarily going to be the same in humans. To the contrary, many vaccines that are effective in animal experiments but showed inadequate protective effects in humans, such as RSV [64] and HIV [65]. Nevertheless, animal experiments are the essential step before trials in human for any vaccines.

## Animal studies of therapeutic agents

More than a dozen anti-CoV drugs have been tested *in vitro* and *in vivo*. Targeting protease and RNA-dependent RNA polymerase (RdRp) are the two of the most effective approaches for inhibition of viral replication. Mycophenolic acid, an inhibitor of inosine-monophosphate dehydrogenase and guanine monophosphate synthesis, displayed a potential anti-MERS-CoV activity in cell culture [66]. However, in an animal study the MERS-CoV infected common marmosets treated with mycophenolate mofetil presented a worse outcome with more severe disease and higher viral loads in necropsied lung than those without treatment [67]. Ribavirin targeting RdRp and Lopinavir-Ritonavir that targeting 3CLpro protease were approved to treat SARS-CoV and MERS-CoV patients, respectively. Nevertheless, these drugs showed limited antiviral effects upon monotherapy and have severe side effects on patients and animals including rhesus macaques, common marmosets and mice. In recent studies, Remdesivir, a broad spectrum antiviral nucleotide prodrug showed a better anti-MERS-CoV activity than Lopinavir and Ritonavir in both in cell culture study and animal studies (hDPP4 transgenic mice and NHPs) [68,69]. Notably, the in vitro anti-SARS-CoV-2 activity of Remdesivir has been demonstrated in a cell culture study [70]. The RBD site of the S protein is the most important target for development of NAbs [58]. These NAbs have been well evaluated in animal models including rhesus macaques and common marmoset, mice, rabbits, ferrets and hamster. However, like the vaccines, abnormal immune responses and ADE effects induced by the antibodies remain to be a serious issue [60].

The interferon signalling is a double-edged sword for therapy of coronavirus infection. On one hand, recombinant interferons showed preventive and antiviral effects in animal models include cynomolgus and rhesus macaques and common marmosets, mice and ferrets at the early stage of SARS-CoV and MERS-CoV infection. Combined action of type I and type III interferon restricts initial replication of SARS-CoV in the lung but fails to inhibit systemic virus spreading in mice [45]. On another hand, delayed administration of interferons induce dysregulated interferon signalling and inflammatory monocyte macrophages responses which cause severe transformation and lethal pneumonia in SARS-CoV infected mice [71]. These experimental findings suggested that a precision intervention time is important for interferon therapy of coronavirus infection.

Hormones that used to restore cytokine storm and other host signalling pathways involved in viral replication might also considered as options to treat coronavirus infection, but only a few of them were evaluated in mice [72]. Moreover, old and new drugs associated with host endosomal, surface and other proteases utilized for viral entry, host receptors mediated viral entry, endocytosis and endosomal acidification also showed ability to inhibit coronavirus infection in cell models, respectively. Nevertheless, unknown in vivo antiviral effects, risk of toxicity and adverse effects might limit their further translation. For example, chloroquine which has been demonstrated able to inhibit viral replication of SARS-CoV and SARS-CoV-2 in vitro, but showed no significant therapeutic effect against SARS-CoV in mice model [73]. Other therapy strategies such as the convalescent-phase plasma still lack mechanism study or detailed in vivo evaluation because the lack of adequate or appropriate animal models. Additionally, testing the antiviral ability of old drugs such as Metformin, Atorvastin, Glitazones, Fibrates and Sartans might be another option at this urgent moment [74]. Clinically, the combined use of several antiviral drugs and host target agents may be more effective than using either modality alone. For example, SARS-CoV patients who received Ribavirin, Lopinavir-Ritonavir and a corticosteroid had lower 21-day SARD and death rates than those who received ribavirin and a corticosteroid [75]. Regards to the patients with severe MERS-CoV infection, viremia was resolved within two days after combined treatment of Ribavirin, Lopinavir-Ritonavir and interferon-α [76].

In sum, we should learn from the lessons of many previous animal experiments. Firstly, a drug with antiviral effects *in vitro* does not necessarily have antiviral effects *in vivo*. Secondly, combination of two or more drugs that have different antiviral mechanisms might achieve a synergistic or an enhanced therapeutic effect. Thirdly, to know the advantages and disadvantages, all the drugs need to be systematically evaluated in several different animal models before clinical study.

## Humanized animal models for studying coronavirus infection

Prevention of and recovery from the coronavirus infection are usually associated with adaptive and innate immunity. The outcomes of the coronavirus infection depend on humoral immune responses such as the subtype and titre of antibody. Although NHPs, mice and other experimental animals can effectively mimic coronavirus infection, the genetic diversity might disturb the interpretation of the possibly different results between human and non-human species. To directly study coronavirus infection in human organ or tissue, humanized animal models are the method of choice, which can also be used for evaluation of coronavirus vaccines and host target agents. In the past decades, mice with one or several human tissues or cells engraftment have been widely used to investigate the pathogenesis of HIV, HBV, HCV, CMV, varicella-zoster virus (VZV) and other important pathogens.

Lung is the main target organ of coronavirus infection. In 2012, Maidji et al. grafted human fetal lung tissues under the kidney capsule of immunodeficient SCID mice [77]. The lung tissues rapidly grew and developed mature structures resembling the normal human lung and were demonstrated to support CMV infection and pathogenesis. In 2017, Wang et al. established a human lung-xenografted mouse model for VZV infection [78]. After VZV infection, viral replication, lung pathogenies and pro-inflammatory cytokine responses were detected in the human lung xenografts. More recently, Wahl et al. reported a humanized mouse with mono human lung engraftment (LoM) or incorporated with human bone marrow, liver and thymus (BLT-L) [79]. The LoM or BLT-L mice supports infection and replication of human pathogens such as MERS-CoV, RSV, CMV and Zika virus [79]. Antigen-specific humoral and T-cell responses were observed in BLT-L mice, suggesting that human lung and immune cell dual chimeric mice might be an ideal humanized animal model for the pathogenesis and immune study of SARS-CoV-2. Due to the abundant expression of hACE2 in multiple organs of human, multiple organs and tissues including liver, heart, intestine, kidney, bladder and immune cells are susceptible to the infection of SARS-CoV-2, suggesting a reason that causes multi-organ failures. Therefore, mice with engraftment of human somatic, progenitor and stem cells, as well as varied human tissues and organoids might be useful to investigate the tropism of SARS-CoV-2.

Comparing to the traditional animal models, the humanized mice provide new options for investigators to directly study the viral infection in human tissues, which is adequate to delineate the tissue tropism and the host-virus interactions. The development of humanized mice is important to improve our fundamental understanding for mechanisms of coronavirus infection and immunopathophysiology. In the future, humanized mice might become a unique tool to obtain the insights into our strategies for developing coronavirus vaccine, early intervention and antiviral therapy.

## Conclusion

The coronaviruses are one of the most important pathogens causing robust respiratory infection, severe cases and deaths in humans. Here, we input key words including coronavirus, animal model, SARS-CoV, MERS-CoV, SARS-CoV-2 (also named 2019-nCoV in the early stage), SARD and COVID-19 to screen the published articles from PubMed. The most related articles have been cited in this review. Recent proceedings and important data of the coronavirus studies are summarized. We suggested that a rapid animal test of SARS-CoV-2 is important to assess the efficiencies of the vaccines, antivirals and the sensitivity of the diagnostic tests. We also debated that a rapid generation of MA viral strains or mice carrying human receptor is a good option for urgent and effective animal studies. In addition, development of humanized animal model might provide a direct infection of coronavirus to human tissue. Taken together, animal models are the fundamental tolls to investigate the viral pathogenesis, to develop vaccines and antiviral drugs.
